# Neural encoding of novel social networks: evidence that perceivers prioritize others’ centrality

**DOI:** 10.1093/scan/nsac059

**Published:** 2022-10-25

**Authors:** Miriam E Schwyck, Meng Du, Pratishta Natarajan, John Andrew Chwe, Carolyn Parkinson

**Affiliations:** Department of Psychology, University of California, Los Angeles, CA 90095, USA; Department of Psychology, University of California, Los Angeles, CA 90095, USA; Silver School of Social Work, New York University, New York, NY 10003, USA; Department of Psychology, New York University, New York, NY 10003, USA; Department of Psychology, University of California, Los Angeles, CA 90095, USA; Brain Research Institute, University of California, Los Angeles, CA 90095, USA

**Keywords:** social networks, fMRI, representational similarity analysis

## Abstract

Knowledge of someone’s friendships can powerfully impact how one interacts with them. Previous research suggests that information about others’ real-world social network positions—e.g. how well-connected they are (centrality), ‘degrees of separation’ (relative social distance)—is spontaneously encoded when encountering familiar individuals. However, many types of information covary with where someone sits in a social network. For instance, strangers’ face-based trait impressions are associated with their social network centrality, and social distance and centrality are inherently intertwined with familiarity, interpersonal similarity and memories. To disentangle the encoding of the social network position from other social information, participants learned a novel social network in which the social network position was decoupled from other factors and then saw each person’s image during functional magnetic resonance imaging scanning. Using representational similarity analysis, we found that social network centrality was robustly encoded in regions associated with visual attention and mentalizing. Thus, even when considering a social network in which one is not included and where centrality is unlinked from perceptual and experience-based features to which it is inextricably tied in naturalistic contexts, the brain encodes information about others’ importance in that network, likely shaping future perceptions of and interactions with those individuals.

## Introduction

When encountering a stranger, the human brain spontaneously encodes specific pieces of information about that person. Information related to inferences of trustworthiness, dominance and other socially relevant characteristics based on facial features is encoded in a set of regions associated with social cognitive processes, often referred to as the default mode network (e.g. Winston *et al.*, 2002; Engell *et al.*, 2007; Gobbini and Haxby, 2007; Wagner *et al.*, 2012; Parkinson et al., 2017; Cao *et al.*, 2020; Su *et al.*, 2021). Recent evidence suggests that people also encode where familiar others sit in their broader social networks, even when there is no task directing their attention to this information (Zerubavel *et al.*, 2015; Parkinson et al., 2017; Peer *et al.*, 2021). Such evidence stems from functional magnetic resonance imaging (fMRI) studies on real-world social networks in which participants viewed images of their fellow network members (e.g. members of the same community). Brain regions associated with mentalizing and attentional allocation encoded how well-connected, or central, the individual was in the participant’s own social network (Zerubavel *et al.*, 2015; Parkinson et al., 2017). Additionally, brain regions implicated in encoding spatial and abstract distances encoded how proximal perceived individuals were in the network (friends, friends-of-friends, friends-of-friends-of-friends, etc.), either to the participant or to each other (Parkinson et al., 2017; Peer *et al.*, 2021). Thus, the human brain appears to prioritize information about familiar others’ positions in one’s real-world social networks and spontaneously retrieves this information when encountering them.

There are many confounding pieces of information, however, that are inextricably tied to where people sit in their social networks. Indeed, when encountering familiar friends, there is a plethora of information immediately available, including personal history, personality and shared experiences, all of which are inherently linked with that person’s social network position. For instance, people who are exceptionally well-connected (e.g. people who have many friends or, in other words, are high in ‘degree centrality’) will likely be seen at more social gatherings and be discussed more frequently and thus become more visually and socially familiar. Furthermore, people are not randomly assigned to positions in their real-world social networks, and thus, there are a variety of factors that may lead individuals to hold their respective places (e.g. more extraverted individuals are likely to have more connections). Recent evidence suggests that, even without first-hand experience with others or knowledge of their personalities, face-based trait impressions (e.g. apparent trustworthiness, warmth and attractiveness) are associated with actual and perceived social network centrality (Alt *et al.*, 2022). That is, naïve observers were able to accurately identify characteristics of others’ social network positions based solely on their facial features. Furthermore, observers’ impressions of targets’ personality traits (again based only on facial features) were linked to where those targets sat in their social network. Thus, familiarity, person knowledge, shared experiences and physical features may systematically covary with real-world network position characteristics, such as relative social distance and social network centrality. Given these potentially confounding factors in real-world social networks, it is difficult to determine if perceivers truly spontaneously encode knowledge of others’ social network positions when encountering them, rather than features that covary with where someone sits in their social network.

Additionally, it is unknown how context shapes the encoding of information related to where people sit in their social network. Different aspects of this information may be more relevant in one context and less so in another. For instance, if the goal is to spread information about an event as quickly as possible, then one would likely seek out the most well-connected individuals who are able to efficiently disseminate the message to as many people as possible. On the other hand, when planning a wedding seating chart, one needs to consider how closely people are connected so that individuals who are nearer to each other in the couple’s social network will be seated together (e.g. a table for a bride’s college friends and another for her partner’s cousins). In the first scenario, an individual’s number of connections, or degree centrality (one measure of how well-connected an individual is in a network), is particularly relevant, while in the second, the geodesic distance between two people (i.e. the number of steps between them in the network) is more relevant. Does this contextual relevancy affect how the mind encodes their social network position when encountering them? It is possible that certain brain regions incorporate the relevancy of information to the current situation and encode information like degree centrality to a greater extent when it is relevant than when it is not. We can thus examine neural patterns elicited by others to shed light on if and where social network information is encoded, as well as how the mind integrates situational factors with person knowledge.

In the current experiment, we taught participants a novel network structure and used fMRI to measure the neural encoding of others’ social network positions in this network. This allowed us to examine the encoding of social network knowledge decoupled from other potential confounding factors that are inherently linked to social network characteristics in real-world contexts. We also systematically varied the contextual relevance of two different facets of others’ network positions: how many friends someone has (degree centrality) and how close people are to one another in the network (relative geodesic distance). Network members (represented by their names and faces) were randomly assigned to positions in a learned social network across participants. In doing so, we were able to dissociate social network position characteristics from confounding variables that exist in real-world social networks and to dissociate degree centrality from the relative geodesic distance. Finally, by using a novel network that participants were not a part of, we were able to test if the brain encodes allocentric (distance between two others) rather than egocentric (distance from oneself) social distance, further isolating social network knowledge from potential feelings of affiliation or preferences for individuals closer to oneself. Through this controlled paradigm, we were able to empirically test if the human brain encodes various aspects of the social network position over and above other aspects of person knowledge, social experiences, familiarity and facial features and to explore how contextual relevancy shapes this encoding.

## Methods

### Participants

Participants were recruited from the University of California, Los Angeles (UCLA) campus via flyers and were required to be fluent in spoken and written English, 18 to 35 years old, right-handed and have no metal in their body. Additionally, participants had to sufficiently learn a social network during session 1 to be eligible for the fMRI session. To reach our target sample size of 30 (determined *a priori*), we recruited 78 participants for session 1, 31 of whom passed (see the Procedure section) and participated in session 2. One subject was excluded due to technical issues with the projection system in the scanner. As such, our final sample size was 30 (13 females, 17 males; ages 18–35 years, *M* = 21.27, standard deviation = 3.33). Participants were paid $15/hour for session 1 and $20/hour for session 2. All recruited participants were consented in accordance with UCLA Institutional Review Board requirements.

### Procedure

**Fig. 1.**
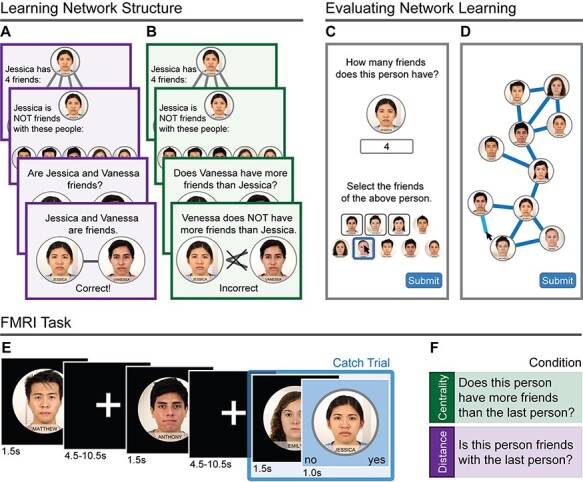
(A and B) Participants learned the social network by viewing each person at the top the screen with their number of friends (degree) and the pictures of those friends at the bottom of the screen. Next, they saw the same individual at the top of the screen with everyone who is not their friend at the bottom in order to ensure that every person is seen the same number of times. (A) On half of the rounds, participants then saw every pair and answered whether or not they were friends. (B) On the other rounds, participants saw each pair and answered whether the person on the right had more friends than the person on the left. They were told if they were correct or not immediately following each trial. After eight rounds, they were evaluated on their knowledge of the network through two tasks. (C) In the first, they reported how many friends and who those friends were for each person, one at a time. (D) In the second task, they drew the network by drawing lines between friends. Participants who knew the network sufficiently completed a practice version of the task they would complete in the scanner. (E) In the fMRI task, they saw each person for 1.5 s followed by jittered fixation time. Randomly throughout each run were a small number of catch trials that were immediately followed by a second image on a blue screen. (F) On half of the runs, participants had to answer if the person on the blue screen had more friends than the last person (centrality condition). On the other half of the rounds, participants answered if the two were friends or not (distance condition). Participants who could successfully complete the scanner task were scheduled for the fMRI session 1–6 days later.

The study was completed in two sessions, one to six days apart. In session 1, participants learned two aspects of a friendship network consisting of 13 friendships among 10 individuals. Specifically, they learned how many friends each individual had (i.e. their degree centrality; Figure 1B) and who was friends with whom (Figure 1A). We then evaluated their knowledge. If participants failed to recall each individual’s degree and friendships with 100% accuracy (Figure 1C) or were less than 70% accurate in their drawing of the network (Figure 1D), then they did not pass the evaluation task, their participation was terminated, and they were paid for their participation. If they did pass the evaluation task, then they completed a practice version of the task they would complete in the scanner (Figure 1E), which required at least 80% accuracy to be eligible to participate in the fMRI session (session 2). We used strict passing thresholds to ensure that participants knew the network well and were able to complete the scanner task. In session 2, participants completed a shortened version of the network learning task before entering the scanner and then completed the fMRI task while in the scanner.

Each node in the network was represented by an emotionally neutral face from the Chicago Face Database (Ma *et al.*, 2015). To aid in distinguishing between people, the shirts were colored and names were added. Images were randomly assigned to the network position across participants. The network was symmetric (Figure 1D) to maximally dissociate degree centrality from the relative distance between people.

#### Session 1

##### Learning the network structure.

Participants learned the two social network features (centrality: number of friends; friendship/distance: friends’ identities) in discrete blocks of a learning paradigm, which was presented using PsychoPy (Peirce, 2009). Participants saw each network member at the top of the screen along with how many friends that person had (i.e. their degree centrality) and who those friends were (Figure 1A and B). To avoid showing well-connected people more often than people who have fewer friends (and thus confounding social network centrality with visual familiarity to participants), this was followed by a trial showing everyone who is not friends with that person.

Next, participants saw each pair in a randomized order and were either asked if the two people were friends with each other (friendship blocks; Figure 1A) or if the person on the right had more friends than the person on the left (centrality blocks; Figure 1B). They were given immediate feedback on whether or not they were correct for a minimum of 0.25 s. This was repeated over eight rounds that were blocked such that the first half of the rounds were of one type and the second half was of the other type, counterbalanced across subjects.

##### Evaluating network learning.

To evaluate how well participants learned each feature of the network, participants were shown each person in the network and asked how many friends they had, followed by who those friends were (Figure 1C). To pass, 100% accuracy was required. Next, participants were asked to draw the full network (Figure 1D). All 10 people were presented, and participants drew lines between those they remembered as friends. To pass this task, participants needed at least 70% accuracy. They were told which ones were wrong (if any) and to fix them before continuing.

##### Practice fMRI task.

To ensure that all participants who participated in session 2 would be able to do the task in the scanner, those who passed the evaluation tasks practiced the fMRI task (presented using PsychoPy) at the end of session 1. During the fMRI task, every trial consisted of one network member being displayed for 1.5 s followed by 4.5–10.5 s of jittered fixation time. Randomly spaced throughout each run were catch trials in which a second image on a blue background was shown immediately after the first for 1 s (Figure 1E). At the beginning of each run, participants were told to answer one of two questions whenever they saw a blue background: (i) ‘does this person have more friends than the last person?’ or (ii) ‘is this person friends with the last person?’ At the end of each round, participants were told how many catch trials they answered correctly, how many they answered incorrectly and how many they missed. Participants needed to reach 80% accuracy in session 1 to be eligible for session 2.

#### Session 2

If participants passed all tasks in session 1, they participated in session 2 one to six days later. During this session, they completed a shortened version of the learning and evaluation tasks described above. During the evaluation task, they were given immediate feedback on each trial and told to correct their mistake to ensure that participants knew the network as well as possible before entering the scanner. Participants then completed eight runs of the fMRI task in the scanner (approximately one hour). Per run, each target person was shown four times as non-catch trials, one time as a catch trial (on a blue screen) and one time as the person shown immediately before the catch trial. Importantly, catch trials were only used to focus participants’ attention on individuals’ relative centrality or distance from others (if they were friends—i.e. separated by a geodesic distance of 1—or not—i.e. separated by a geodesic distance greater than 1). We only analyzed the four occurrences of each image that were not part of a catch trial to test if people encoded social network information even when they were not asked about it directly.

### FMRI data acquisition

MRI data were collected on a Siemens 3-Tesla Prisma Fit MRI Scanner with a 32-channel head coil. Functional scans were obtained using a gradient echo sequence with 64 interleaved slices (2.0 mm isotropic voxels, repetition time (TR) = 750 ms, echo time (TE) = 35 ms, flip angle = 52° and field of view (FOV) = 184 mm). Participants used a 2-button response box to make choices during the task. For each subject, two echo planar field maps were obtained after functional scans began in order to correct for the effects of field inhomogeneity. Finally, a T1-weighted (T1w) MPRAGE sequence (1 mm isotropic voxels, 208 slices, TR = 1900 ms, TE = 2.48 ms, flip angle = 9° and FOV = 256 mm) was acquired after the field maps.

### FMRI analyses

#### Image preprocessing

Preprocessing was performed using fMRIPrep 1.4.0 (Esteban et al., 2019), which is based on Nipype 1.2.0 (Gorgolewski et al., 2019). The preprocessing descriptions provided here are taken from the recommended citation boilerplate text generated by fMRIPrep (released under a CC0 license with the intention that researchers reuse the text to facilitate clear, consistent descriptions of preprocessing steps, thereby enhancing reproducibility).

##### Anatomical data preprocessing.

The T1w image was corrected for intensity nonuniformity with N4BiasFieldCorrection, distributed with ANTs 2.1.0 (Avants *et al.*, 2008) and used as the T1w reference throughout the workflow. The T1w reference was skull-stripped with a Nipype implementation of the antsBrainExtraction.sh workflow, using OASIS30ANTs as a target template. Brain tissue segmentation of cerebrospinal fluid (CSF), white matter (WM) and gray matter was performed on the brain-extracted T1w using fast (Smith *et al.*, 2004). Brain surfaces were reconstructed using recon-all (FreeSurfer 6.0.0; Dale *et al.*, 1999).

##### Functional data preprocessing.

For each of the eight blood oxygen level-dependent (BOLD) runs per subject, the following preprocessing was performed. First, a reference volume and its skull-stripped version were generated. A deformation field to correct for susceptibility distortions was estimated based on two echo planar imaging references with opposing phase-encoding directions, using 3dQwarp in AFNI (Cox, 1996; Cox and Hyde, 1997). Based on the estimated susceptibility distortion, an unwarped BOLD reference was calculated for a more accurate co-registration with the anatomical reference. The BOLD reference was co-registered to the T1w reference using bbregister in FreeSurfer with nine degrees of freedom to account for distortions remaining in the BOLD reference. Head motion parameters with respect to the BOLD reference (transformation matrices; six corresponding rotation and translation parameters) were estimated before any spatiotemporal filtering using FSL’s mcflirt (Jenkinson *et al.*, 2002). The BOLD time series were resampled onto their original, native space by applying a single, composite transform to correct for head motion and susceptibility distortions. The first six volumes of each scan were removed from data prior to subsequent analyses.

#### First-level analysis

We fit a general linear model within each catch trial condition (focused on either degree centrality or friendship) of the fMRI data using Nistats (Abraham et al., 2014) to estimate the BOLD response evoked for each of the 10 nodes in the network (represented by different images across participants). The following confounding variables were included in the model as nuisance regressors: three translational motion parameters, three rotational motion parameters, three global signals extracted within the CSF, WM and whole-brain mask. All regressors of interest were convolved with a Glover hemodynamic response function. The t-statistic maps (i.e. maps of beta coefficients divided by their standard error estimates) resulting from these analyses were used for subsequent pattern similarity analyses.

##### Overall encoding of social network position characteristics.

**Fig. 2.**
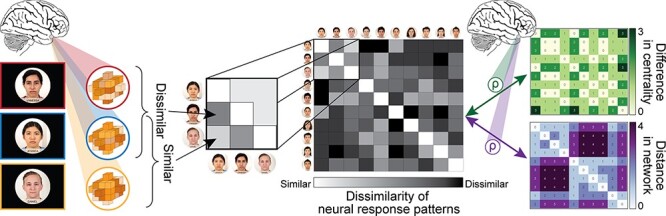
Using only the non-catch trials in the fMRI task, patterns of neural activation were extracted for each network member in a searchlight procedure. At each searchlight center (i.e. each voxel), the response pattern within a sphere centered on that voxel was extracted for each person seen by the participant while in the scanner. The Euclidean distances between each pattern were then calculated and arranged into a RDM in which each row and column are associated with a network member and the corresponding cell is the dissimilarity in patterns of activity elicited by those two people. This RDM was then Spearman rank–correlated with RDMs representing the difference in network members’ centralities (top right matrix) and the distance between people in the network (bottom right matrix). At the group level, we tested where each correlation coefficient (*ρ*) at each point in the brain (i.e. at each searchlight center) was significantly greater than zero.

We tested if and where each facet of the network position was encoded throughout the fMRI task, regardless of the condition. To do so, we first averaged the *t*-maps from the two conditions, resulting in one overall distributed neural response pattern for each target person participants encountered in the study. Using a searchlight procedure, we iteratively extracted the multi-voxel pattern of *t*-values evoked by each target person within ‘spheres’ (radius = 4 voxels) centered at each voxel. We conducted representational similarity analysis (RSA) using these neural response patterns (Kriegeskorte *et al.*, 2008), which allowed us to compare neural representations to models based on each facet of the social network position (degree centrality and social distance) to test if a brain region encoded that particular feature (Figure 2; Weaverdyck *et al.*, 2020).

This was achieved through the creation and comparison of representational dissimilarity matrices (RDMs). First, we created the model centrality RDM in which each row and column were associated with an individual in the network and the corresponding cell in the matrix was the absolute value of the difference in degree centrality between those two individuals (Figure 2, top right matrix). Similarly, we created a model distance RDM in which each cell represented the geodesic distance between two individuals in the network (Figure 2, bottom right matrix).

We then compared these model RDMs with neural RDMs. To create the neural RDMs, we calculated the Euclidean distance between the neural patterns elicited by different nodes. The Euclidean distance was used as it reflects differences in both overall response magnitudes and in topological response patterns between conditions. For results from parallel analyses using Pearson correlations, which reflect differences in topological response patterns only, see the Supplementary Materials (Figure S1 and Tables S1 and S2). That is, each cell of the neural RDMs reflected how similar a brain region represented the people corresponding to that cell’s row and column. Next, to determine the extent to which each facet of social network knowledge was encoded (independent of the other), we calculated the Spearman rank correlation coefficient, *ρ*, between the lower off-diagonal triangles of the neural RDMs and each model RDM (Figure 2). (Spearman correlation was used instead of Pearson correlation because it does not assume a linear relationship between the neural and model RDMs.) In other words, we tested if similarity in neural representations was correlated with similarities in degree centrality or proximity in the network. The correlation coefficients were then mapped back onto the central voxel of the searchlight sphere. The two resulting whole-brain maps demonstrated the extent to which distributed neural response patterns in each region (area surrounding each voxel) reflected the degree centrality and the relative social distance of the people being viewed.

##### Encoding based on contextual relevancy.

How does context shape the encoding of this information? To begin to answer this question, we conducted the same analysis described above within each of the conditions: the centrality condition when participants were focused on individuals’ degree centralities, and the distance condition when participants were focused on individuals’ relationships (i.e. their ‘degrees of separation’ from one another). To test if degree centrality was encoded more when it was relevant than when it was irrelevant, we subtracted the correlation coefficients between the neural RDMs and the centrality model RDM in the irrelevant condition from the relevant condition (i.e. centrality-relevant condition > distance-relevant condition). We ran the same analysis for distance, testing where the correlation coefficient between the neural RDM and the distance model RDM was higher in the relevant condition than in the irrelevant condition (i.e. distance-relevant condition > centrality-relevant condition).

#### Second-level analysis

All first-level analyses were conducted in participants’ T1w space. For group-level analyses, we transformed individuals’ first-level maps to The ICBM 152 Nonlinear Asymmetrical template version 2009c (Collins et al., 1999; Fonov et al., 2011) space using ANTs and the mapping generated by fMRIPrep. All first-level maps underwent smoothing (6 mm full width at half maximum Gaussian kernel). To determine where the brain encoded centrality or distance at the group level, we ran nonparametric permutation testing (10 000 iterations) using FSL’s randomise function (Winkler et al., 2014) within a 5-mm-dilated brain mask, with 10-mm variance smoothing. Results underwent threshold-free cluster enhancement (TFCE) (Smith and Nichols, 2009) to correct for multiple comparisons.

#### Parcellation analyses

In addition to the whole-brain searchlight analyses described above, we ran the same analyses in each parcel of the 200-region Schaefer parcellation (Schaefer et al., 2018) to test for convergence. We conducted both searchlight and parcellation analyses because the searchlight approach provides continuous statistical maps of social network encoding, but defines regions as artificial spheres that are unlikely to resemble the size or shape of functionally or anatomically defined brain regions, which could lead, for example, to collapsing response patterns across functionally distinct areas. The parcellation approach, however, results in a coarser map of encoding, but defines regions based on their functional response profiles (or anatomy, depending on the parcellation chosen). We transformed the parcellation to each participant’s T1w space using the mapping generated by fMRIPrep and ANTs. We extracted the patterns of *t*-values within each region to create the neural RDMs. The correlations between the neural and model RDMs were then mapped back onto each region. To determine if a region encoded centrality or distance at the group level, we conducted one-sample one-sided t-tests (ρ > 0) within each region in R (Version 3.6.1; R Core Team, 2018). *P*-values were corrected for multiple comparisons across the 200 parcels using false discovery rate (FDR) correction.

## Results

### Neural encoding of degree centrality

**Fig. 3.**
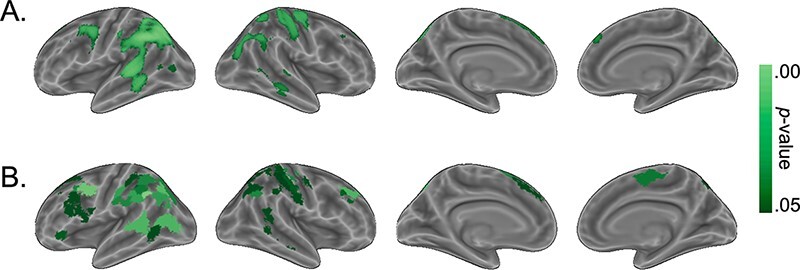
Regions that showed significant encoding of degree centrality across conditions as measured by correlations between neural RDMs and the degree centrality RDM. (A) Results using a searchlight procedure with a four-voxel radius. All *p*-values from searchlight-based analyses were corrected for multiple comparisons using TFCE. (B) Results using the 200-region Schaefer parcellation. All *p*-values from parcellation-based analyses were FDR-corrected for multiple comparisons. Only regions that surpass a corrected threshold of *p* < .05 are shown.

Using RSA, we tested if and where two facets of the social network position (degree centrality and social distance from others) were encoded overall (i.e. across conditions). First, we tested which regions encoded network members’ degree centrality (i.e. how many friends they had). Results from the searchlight analysis show significant encoding of others’ degree centrality bilaterally in large swaths of cortex around the temporoparietal junction (TPJ), superior parietal lobule, inferior parietal lobule, superior temporal gyrus (STG) and middle temporal gyrus (Figure 3A; Table 1). We found convergent results using the Schaefer parcellation (Figure 3B; Table 2).

**Table 1.** Searchlight clusters that encoded degree centrality

General region	*N* voxels	Peak *t-*value	Peak coordinates (*x*, *y*, *z*)	Center of gravity (*x*, *y*, *z*)
Posterior lateral temporal cortex, posterior parietal cortex, and occipital cortex	36 194	5.36	(63.5, −28.5, −8.5)	(−4. 3, −56, 34.9)
Left dorsolateral prefrontal cortex	865	4.12	(−38.5, 15.5, 59.5)	(−44.9, 12.1, 50.6)
Left dorsomedial prefrontal cortex	128	3.64	(−4.5, 11.5, 61.5)	(−3.67, 9.64, 61.4)

Note. Significant clusters from the searchlight analysis (TFCE-corrected, *p *< .05). General regions are named based on the approximate location of the cluster. Localization of clusters is depicted more precisely in Figure 3A.

**Table 2.** Parcels that encoded degree centrality

General region	Network	Index	*ß*	*t*	d*f*	*p*
Left posterior parietal cortex	Control	61	0.15	4.66	29	.006**
Left premotor cortex	Control	70	0.16	4.20	29	.012*
Left posterior parietal cortex	Dorsal attention	37	0.11	3.77	29	.019*
Left posterior parietal cortex	Control	62	0.15	3.85	29	.019*
Left superior temporal cortex	Default	78	0.12	3.57	29	.022*
Right superior frontal cortex	Default	196	0.10	3.56	29	.022*
Left lateral occipital cortex	Visual	8	0.08	3.50	29	.022*
Left posterior parietal cortex	Dorsal attention	34	0.11	3.32	29	.030*
Left posterior parietal cortex	Salience/ventral attention	46	0.12	3.28	29	.030*
Left posterior parietal cortex	Default	82	0.11	3.22	29	.031*
Left posterior parietal cortex	Dorsal attention	36	0.10	3.00	29	.037*
Left dorsolateral prefrontal cortex	Default	93	0.11	3.00	29	.037*
Left medial premotor cortex	Default	95	0.10	3.04	29	.037*
Right somatomotor cortex	Somatomotor	127	0.11	3.02	29	.037*
Right posterior parietal cortex	Default	184	0.10	3.09	29	.037*
Left posterior parietal cortex	Salience/ventral attention	45	0.10	2.97	29	.037*
Left lateral occipital cortex	Dorsal attention	33	0.13	2.93	29	.038*
Left posterior parietal cortex	Control	63	0.12	2.87	29	.040*
Right somatomotor cortex	Somatomotor	126	0.09	2.88	29	.040*
Right premotor cortex	Control	175	0.09	2.80	29	.045*
Left ventrolateral prefrontal cortex	Default	85	0.08	2.73	29	.046*
Left dorsomedial prefrontal cortex	Default	91	0.10	2.74	29	.046*
Right somatomotor cortex	Somatomotor	124	0.09	2.75	29	.046*
Left middle temporal gyrus	Dorsal attention	32	0.08	2.57	29	.048*
Left posterior parietal cortex	Dorsal attention	35	0.06	2.52	29	.048*
Left ventrolateral prefrontal cortex	Salience/ventral attention	50	0.09	2.67	29	.048*
Left dorsolateral prefrontal cortex	Control	69	0.09	2.55	29	.048*
Left dorsomedial prefrontal cortex	Default	92	0.09	2.53	29	.048*
Left dorsolateral prefrontal cortex	Default	94	0.10	2.68	29	.048*
Right somatomotor cortex	Somatomotor	125	0.07	2.60	29	.048*
Right somatomotor cortex	Somatomotor	128	0.10	2.52	29	.048*
Right somatomotor cortex	Somatomotor	130	0.07	2.51	29	.048*
Right posterior parietal cortex	Dorsal attention	137	0.10	2.55	29	.048*
Right precuneus	Dorsal attention	140	0.09	2.53	29	.048*
Right middle temporal gyrus and superior temporal sulcus	Salience/ventral attention	148	0.09	2.62	29	.048*
Right posterior parietal cortex	Control	166	0.09	2.52	29	.048*
Right middle temporal gyrus and superior temporal sulcus	Default	188	0.09	2.52	29	.048*
Right posterior parietal cortex	Control	167	0.08	2.50	29	.049*
Right posterior parietal cortex	Dorsal attention	142	0.08	2.48	29	.049*
Right posterior parietal cortex	Dorsal attention	139	0.09	2.47	29	.050*

Note. Indices and network names are provided by the database corresponding to the Schaefer *et al.* (2018) parcellation. General regions are named based on the approximate location of parcel. All *p*-values are corrected for multiple comparisons using FDR-based correction. *p < .05; **p < .01.

### Neural encoding of the social distance

**Fig. 4.**
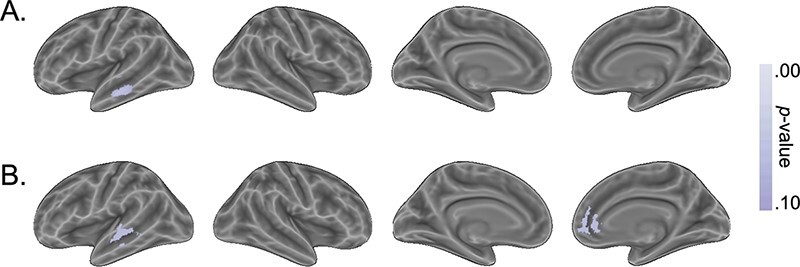
Regions that trended toward encoding the distance between network members. Note that there were no significant regions after correcting for multiple comparisons. (A) Results using a searchlight procedure with a four-voxel radius. All *p*-values from searchlight-based analyses were corrected for multiple comparisons using threshold-free cluster enhancement. (B) Results using the 200-region Schaefer parcellation. All *P*-values from parcellation-based analyses were FDR-corrected for multiple comparisons. Only regions that surpass a corrected threshold of *p* < .1 are shown.

Next, we tested if and where the social distance between network members was encoded. We did not find any significant (*p *< .05) encoding of the distance. However, we did find that the overall encoding of targets’ social distances to one another was trending (p < .10) in a 520-voxel cluster in the left middle temporal gyrus [peak value = 4.59, peak coordinates = (−62.5, −24.5, −10.5), center of gravity = (−61.2, −24.4, −11.7)] in the searchlight analysis (Figure 4A) and, in the parcellation-based analysis, in the right medial prefrontal cortex (mPFC) [*ρ* = 0.07, *t*(29) = 3.53, *p* = .070] and anterior STG [*ρ* = 0.09, *t*(29) = 3.58, *p* = .070] (Figure 4B). Since none of these results reached significance, these findings must be interpreted with caution and future research is required to confirm and clarify them.

### Effects of contextual relevancy on the encoding of social network information

Lastly, we tested if and where contextual goals modulated the encoding of centrality. That is, we examined if centrality was encoded *more* when it was relevant than when it was irrelevant. In both the searchlight and parcellation analyses, we did not find any regions that survived correction for multiple comparisons. Similarly, we tested if and where contextual goals modulated the encoding of the distance. We did not find any regions that significantly encoded the distance more when it was relevant than when it was irrelevant.

## Discussion

In this study, we tested if and where the human brain encodes information about others’ social network positions when that information is disassociated from other features that covary with it in real-world contexts. Specifically, we tested if and where relative degree centrality and social distance were tracked by the brain when viewing others’ faces. To decouple these social network features from other typically confounding types of information (e.g. trait impressions, person knowledge, visual characteristics, familiarity and memories), we taught participants a new social network where network members’ identities were randomly assigned to positions in the network.

We found that degree centrality was robustly encoded in broad regions surrounding the superior and inferior parietal lobules, TPJ and superior and middle temporal gyri. That is, the brain prioritized information regarding others’ centrality in the social network even though the participant was not directly involved in that network and even when other facets of the social network position were more relevant. This suggests that social network centrality may be chronically important to monitor. Indeed, measures of social network centrality capture the importance of a person in a social network and can be considered to comprise a facet of social status (Weaverdyck and Parkinson, 2018; Basyouni and Parkinson, 2022). Here, we found that centrality was encoded in regions that support attentional modulation (Kastner and Ungerleider, 2000; Corbetta and Shulman, 2002; Uncapher and Wagner, 2009) and social cognitive processes, such as understanding others’ mental states (Van Overwalle and Baetens, 2009; Tamir *et al.*, 2016; Morelli *et al.*, 2018). Additionally, our results overlap significantly with previous findings regarding the spontaneous encoding of other’s centrality in their real-world social networks (Zerubavel *et al.*, 2015; Parkinson et al., 2017). Thus, it may be that social network centrality modulates one’s overall attention towards others, because it signals those individuals’ importance in the community and/or as people who are particularly valuable to attend to for ascertaining group norms (Paluck and Shepherd, 2012; Basyouni and Parkinson, 2022). This may also increase attention to and consideration of high-status individuals’ mental states, which could then shape downstream thoughts and behaviors. Given the potential implications for social influence and reputation management (Weaverdyck and Parkinson, 2018), future research should test these possibilities by examining how social network–based status shapes how much perceivers attend to others and to what they appear to be thinking.

While we did not find any significant encoding of the relative distance in the network, we found a trend suggesting that allocentric distances may be encoded in aspects of the lateral temporal cortex and mPFC. Importantly, previous research has primarily focused on if and where the egocentric distance (distance from oneself) is encoded in the brain (Zerubavel *et al.*, 2015; Parkinson et al., 2017). Here, however, we taught participants a new network and tested the extent to which allocentric distance (distance between others) was encoded. Because participants were not members of this network, we cannot directly test the extent to which egocentric distance was encoded in this controlled setting and are thus unable to directly compare our results to the previous literature examining egocentric distance; given that allocentric distance is distinct from egocentric distance, its neural representation may differ. It could simultaneously be that allocentric distance—particularly in a novel network in which one has no part—is encoded less robustly than more self-relevant information (e.g. egocentric distance), and we were not sufficiently powered to detect it in our paradigm. Thus, it is unclear if our trending result is due to a lack of effect or a lack of power in our sample to detect the encoding of allocentric distance.

One reason to suspect that the trending results in the lateral temporal cortex and mPFC are due to a lack of power is that these areas overlap with and neighbor regions that are known to support person models (Wagner *et al.*, 2012; Hassabis *et al.*, 2014; Welborn and Lieberman, 2015; Wang *et al.*, 2017). It could be, then, that people who are close to each other in the network are assumed to be more similar to each other due to phenomena such as homophily (i.e. the tendency for similar others to become friends) and social influence (Son *et al.*, 2021; Schwyck *et al.*, 2022). This would result in the brain representing more proximate individuals in a network as more similar, which is consistent with the trending results. Second, there is recent evidence that allocentric distance in one’s real-world social media network is encoded in the default mode network, including similar regions to those found in the present study, and that allocentric distance is encoded distinctly from egocentric distance (Peer *et al.*, 2021). Thus, there are several possible reasons why the current results differ from previous findings. First, previous findings studying real-world social networks may partially reflect *similarity* of person knowledge or associated memories. Second, egocentric distance might be encoded more robustly than allocentric distance because it connotes self-relevance. Finally, egocentric and allocentric social distances may be qualitatively different types of information that are processed differently in the brain.

The current study implemented a controlled task in which participants learned a new pattern of relationships. This was an intentional departure from previous research that used participants’ real-world social networks, but where social network positions were inextricably linked to other types of social knowledge (e.g. memories) and perception (e.g. visual familiarity and face-based trait impressions; Zerubavel *et al.*, 2015; Parkinson et al., 2017; Peer *et al.*, 2021). These previous studies along with the current research present strong evidence that the human brain prioritizes the recall of social network knowledge when encountering others.

Future research is needed to replicate these findings and extend this work in several ways. In the current study, participants were not directly part of the learned social network and the task directed people to specific network features. This limited us to examining the encoding of allocentric (and not egocentric) social distances and may have rendered participants less likely to call to mind information about others’ network positions when viewing them than they would be in a more personally relevant social network. Future research should work to further disentangle how egocentric and allocentric distances are encoded, how contextual goals modulate the encoding of social networks in which the participant is included and if this information is spontaneously encoded (i.e. even when the task does not direct participants’ attention towards this information), as previous research suggests (Parkinson et al., 2017). Additionally, future research should examine how these facets of social network positions are neurally encoded for different types of relationships other than friendships (e.g. kinship and work hierarchies) and how other measures of node importance (e.g. eigenvector centrality and betweenness centrality) are encoded and used to facilitate inferences about other traits (e.g. competence), likely shaping downstream processes and behaviors. Finally, it is important to examine if and how these phenomena differ across cultures and age groups and as a function of other individual differences (e.g. in patients with disorders characterized by atypical social functioning). Such studies (necessitating much larger sample sizes; Marek *et al.*, 2022) could focus on understanding how the encoding and application of network knowledge differ in perceivers with different social cognitive abilities and from different backgrounds. These expansions of the current work would provide valuable insight into how the brain represents, uses and integrates information about the social networks in which everyone is embedded.

As it has been said time and time again, humans are social animals. The people with whom we regularly interact do not exist in a vacuum, but rather, in the broader context of our social networks. As such, our perceptions of others are defined not only by our impressions and knowledge of them as individuals but also by the patterns of social relationships that surround them. Understanding this social structure is impactful in everyday life, yet it is not well-understood. It is important to characterize how healthy brains support the capacity to learn and represent social networks to understand how such social processes may be compromised in disorders characterized by deficits in social cognition and behavior. One’s ability to learn and process new social network information, and apply it in different contexts, likely has serious consequences for downstream behavioral interactions in all aspects of one’s social life. Here, we found evidence that the human brain prioritizes specific aspects of social network knowledge that signal the relative importance of others in a community.

## Supplementary Material

nsac059_Supp

## Data Availability

The code and data underlying this article will be shared on reasonable request to the corresponding author.
