# Supplementary material for: Neural encoding of novel social networks: evidence that perceivers prioritize others’ centrality
Source: Soc Cogn Affect Neurosci. 2022 Oct 25;18(1):nsac059. doi: 10.1093/scan/nsac059 (PMC9949589; doi:10.1093/scan/nsac059)
Supplement: nsac059_Supp [file nsac059_supp.zip › scan-22-119-File008.docx]

**Supplement for**

***Neural encoding of novel social networks:
Evidence that perceivers prioritize others’ centrality***

Miriam E. Schwyck, Meng Du, Pratishta Natarajan, John Andrew Chwe, Carolyn Parkinson

Figure S1

Neural Encoding of Degree Centrality Using Pearson Correlation


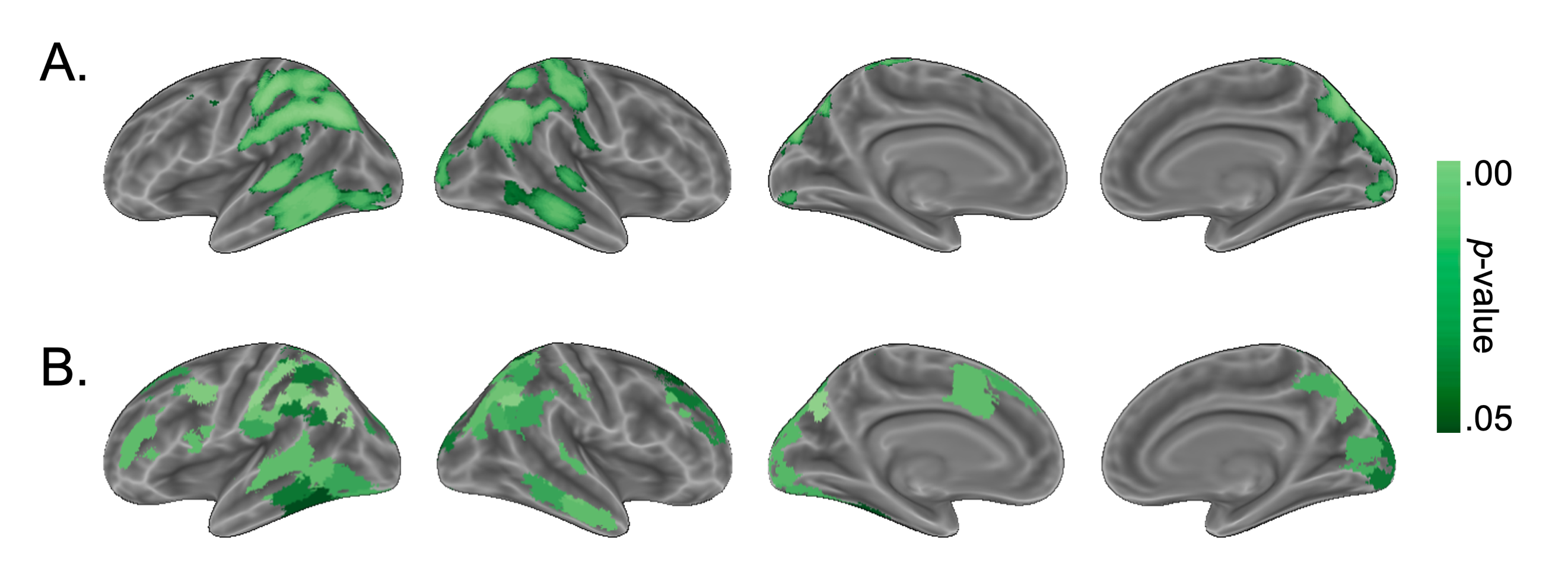


*Note*. Regions that showed significant encoding of degree centrality across conditions as measured by correlations between neural RDMs (comprised of Pearson correlation coefficients) and the degree centrality RDM. (A) Results using a searchlight procedure with a four-voxel radius. All searchlight *p*-values were corrected for multiple comparisons using threshold-free cluster enhancement. (B) Results using the 200-region Schaefer parcellation. All parcellation *p*-values were FDR-corrected for multiple comparisons. Only regions that surpass a corrected threshold of *p* < .05 are shown.

Table S1

Searchlight Clusters That Encoded Degree Centrality Using Pearson Correlation

| General Region | *N* Voxels | Peak *t-*Value | Peak Coordinate (*x, y, z*) | Center of Gravity (*x, y, z*) |
| --- | --- | --- | --- | --- |
| Posterior Lateral Temporal Cortex, Posterior Parietal Cortex, and Occipital Cortex | 36,194 | 5.36 | (63.5, -28.5, -8.5) | (-4.3, -56, 34.9) |
| Left Dorsolateral Prefrontal Cortex | 865 | 4.12 | (-38.5, 15.5, 59.5) | (-44.9, 12.1, 50.6) |
| Left Dorsomedial Prefrontal Cortex | 128 | 3.64 | (-4.5, 11.5, 61.5) | (-3.67, 9.64, 61.4) |

*Note.* Significant clusters from the searchlight analysis (TFCE-corrected, *p* < .05). General regions are named based on approximate location of the cluster. Localization of clusters is depicted more precisely in Fig. 3A.

Table S2

Parcels That Encoded Degree Centrality Using Pearson Correlation

| General Region | Network | Index | ß | *t* | df | *p* |
| --- | --- | --- | --- | --- | --- | --- |
| Left Posterior Parietal Cortex | Control | 71 | 0.14 | 5.39 | 29 | .001*** |
| Left Posterior Parietal Cortex | Control | 61 | 0.16 | 4.89 | 29 | .002** |
| Left Posterior Parietal Cortex | Default | 82 | 0.13 | 4.75 | 29 | .002** |
| Left Posterior Parietal Cortex | Dorsal Attention | 34 | 0.16 | 4.49 | 29 | .002** |
| Left Posterior Parietal Cortex | Control | 62 | 0.15 | 4.44 | 29 | .002** |
| Left Posterior Parietal Cortex | Dorsal Attention | 35 | 0.15 | 4.15 | 29 | .004** |
| Right Posterior Parietal Cortex | Default | 184 | 0.14 | 4.10 | 29 | .004** |
| Left Premotor Cortex | Control | 70 | 0.14 | 3.87 | 29 | .007** |
| Left Posterior Parietal Cortex | Control | 63 | 0.15 | 3.68 | 29 | .010** |
| Right Precuneus | Dorsal Attention | 140 | 0.13 | 3.67 | 29 | .010** |
| Right Middle Temporal Gyrus and Superior Temporal Sulcus | Default | 188 | 0.12 | 3.56 | 29 | .012* |
| Left Posterior Parietal Cortex | Salience/Ventral Attention | 45 | 0.13 | 3.49 | 29 | .012* |
| Right Posterior Parietal Cortex | Control | 167 | 0.13 | 3.48 | 29 | .012* |
| Left Somatomotor Cortex | Somatomotor | 25 | 0.11 | 3.44 | 29 | .013* |
| Left Somatomotor Cortex | Somatomotor | 23 | 0.13 | 3.38 | 29 | .014* |
| Left Middle Temporal Gyrus | Dorsal Attention | 32 | 0.11 | 3.28 | 29 | .016* |
| Left Dorsolateral Prefrontal Cortex | Control | 68 | 0.10 | 3.23 | 29 | .016* |
| Right Lateral Occipital Cortex | Visual | 115 | 0.09 | 3.22 | 29 | .016* |
| Right Premotor Cortex | Control | 175 | 0.11 | 3.24 | 29 | .016* |
| Right Middle Temporal Sulcus | Default | 186 | 0.08 | 3.26 | 29 | .016* |
| Right Medial Occipital Cortex | Visual | 114 | 0.12 | 3.19 | 29 | .016* |
| Left Superior Temporal Cortex | Default | 78 | 0.11 | 3.17 | 29 | .016* |
| Left Ventrolateral Prefrontal cortex | Salience/Ventral Attention | 50 | 0.12 | 3.09 | 29 | .017* |
| Left Cingulate Cortex | Salience/Ventral Attention | 52 | 0.1 | 3.06 | 29 | .017* |
| Left Middle Temporal Gyrus and Superior Temporal Sulcus | Default | 77 | 0.08 | 3.11 | 29 | .017* |
| Right Somatomotor Cortex | Somatomotor | 124 | 0.10 | 3.08 | 29 | .017* |
| Right Somatomotor Cortex | Somatomotor | 128 | 0.13 | 3.06 | 29 | .017* |
| Right Posterior Parietal Cortex | Control | 177 | 0.10 | 3.12 | 29 | .017* |
| Left Occipital Pole | Visual | 7 | 0.13 | 3.02 | 29 | .018* |
| Right Posterior Parietal Cortex | Dorsal Attention | 142 | 0.11 | 3.02 | 29 | .018* |
| Right Precuneus Cortex | Default | 200 | 0.10 | 2.98 | 29 | .019* |
| Left Dorsomedial Prefrontal Cortex | Default | 92 | 0.10 | 2.95 | 29 | .019* |
| Right Posterior Parietal Cortex | Control | 166 | 0.11 | 2.89 | 29 | .022* |
| Right Posterior Parietal Cortex | Default | 182 | 0.09 | 2.87 | 29 | .022* |
| Left Posterior Parietal Cortex | Dorsal Attention | 38 | 0.12 | 2.86 | 29 | .022* |
| Right Calcarine Cortex | Visual | 109 | 0.10 | 2.84 | 29 | .022* |
| Left Fusiform Gyrus | Visual | 2 | 0.12 | 2.82 | 29 | .023* |
| Left Dorsomedial Prefrontal Cortex | Default | 91 | 0.09 | 2.80 | 29 | .024* |
| Right Inferior Temporal Gyrus and Middle Temporal Sulcus | Control | 168 | 0.10 | 2.78 | 29 | .024* |
| Left Lateral Occipital Cortex | Visual | 3 | 0.09 | 2.77 | 29 | .024* |
| Left Somatomotor Cortex | Somatomotor | 19 | 0.09 | 2.71 | 29 | .027* |
| Right DorsoAttn_Post | Dorsal Attention | 141 | 0.08 | 2.68 | 29 | .028* |
| Left Posterior Parietal Cortex | Dorsal Attention | 37 | 0.10 | 2.69 | 29 | .028* |
| Left Dorsolateral Prefrontal Cortex | Default | 93 | 0.11 | 2.68 | 29 | .028* |
| Right Posterior Parietal Cortex | Default | 183 | 0.09 | 2.64 | 29 | .028* |
| Left Dorsolateral Prefrontal Cortex | Default | 94 | 0.09 | 2.64 | 29 | .028* |
| Left Lateral Occipital Cortex | Visual | 14 | 0.11 | 2.64 | 29 | .028* |
| Right Dorsolateral Prefrontal Cortex | Control | 174 | 0.07 | 2.57 | 29 | .032* |
| Right Occipital Pole | Visual | 112 | 0.10 | 2.55 | 29 | .034* |
| Left Somatomotor Cortex | Somatomotor | 27 | 0.08 | 2.52 | 29 | .035* |
| Left Posterior Parietal Cortex | Dorsal Attention | 36 | 0.09 | 2.45 | 29 | .040* |
| Left Posterior Parietal Cortex | Salience/Ventral Attention | 46 | 0.10 | 2.4 | 29 | .041* |
| Left Inferior Temporal Gyrus and Middle Temporal Sulcus | Control | 64 | 0.09 | 2.39 | 29 | .041* |
| Left Posterior Parietal Cortex | Default | 81 | 0.10 | 2.40 | 29 | .041* |
| Right Occipital Pole | Visual | 106 | 0.07 | 2.41 | 29 | .041* |
| Right Superior Frontal Cortex | Default | 196 | 0.09 | 2.39 | 29 | .041* |
| Right Superior Frontal Cortex | Default | 197 | 0.10 | 2.40 | 29 | .041* |
| Right Posterior Parietal Cortex | Dorsal Attention | 144 | 0.07 | 2.44 | 29 | .041* |
| Left Fusiform Cortex | Dorsal Attention | 31 | 0.08 | 2.32 | 29 | .047* |

*Note.* Indices and network names are provided by the database corresponding to the Schaefer et al. (2018) parcellation. General regions are named based on approximate location of parcel. All *p*-values are corrected for multiple comparisons using FDR-based correction. * *p* < .05; ** *p* < .01; *** *p* < .001
